# Microbiota-gut-brain axis: relationships among the vagus nerve, gut microbiota, obesity, and diabetes

**DOI:** 10.1007/s00592-023-02088-x

**Published:** 2023-04-14

**Authors:** Susanna Longo, Stefano Rizza, Massimo Federici

**Affiliations:** Department of Systems Medicine, University of Rome Tor Vergata, Via Montpellier 1, 00133 Rome, Italy

**Keywords:** Diabetes, Diabesity, GLP-1, Gut microbiota, Obesity, Vagus nerve

## Abstract

**Aims:**

The purpose of this review is to explore the interconnected pathways of the microbiota-gut-brain axis (MGBA), focusing on the roles of the vagus nerve and glucagon like peptide-1 in appetite control, and in the development of obesity and diabetes.

**Methods:**

Type 2 diabetes mellitus (T2DM) and obesity are metabolic disorders whose prevalence has significantly increased in recent decades and is expected to increase every year, to pandemic proportions. These two pathologies often coexist and have substantial public health implications. The term “diabesity” defines the pathophysiological connection between overweight and T2DM. The gut microbiota affects many aspects of the host. Beyond the regulation of intestinal functions and the activation of immune responses, the gut microbiota plays a role in central nervous system functions (i.e., mood, and psychiatric conditions associated with stress and memory) and is a central regulator of metabolism and appetite.

**Results:**

The MGBA involves pathways such as the autonomic and enteric nervous systems, the hypothalamic– pituitary–adrenal axis, the immune system, enteroendocrine cells, and microbial metabolites. Notably, the vagus nerve plays an essential role in eating behavior by modulating appetite and learning nutritional preferences.

**Conclusions:**

Because of its enteroendocrine cell-mediated interaction with the gut microbiota, the vagus nerve may provide a potential pathway through which gut microorganisms influence host feeding behavior and metabolic control of physiological and pathological conditions.

## Introduction

The connections among the central nervous system (CNS), the gut and the gut microbiota are central to microbiota-host synergy and explain how the gut microbiota influences several aspects of host behavior [1]. These connections form the microbiota-gut-brain axis (MGBA). Over the past decade, a strong association between changes in microbiota composition (i.e., dysbiosis) and various host pathological conditions has been found [2]. Notably, the gut microbiome has emerged as a targetable organ influencing the development of some metabolic diseases. This aspect is important, given the ever-increasing global prevalence of obesity and type 2 diabetes mellitus (T2DM). With their spread, obesity and T2DM pose major economic and health burdens [3]. In 2016, the World Health Organization estimated that more than 650 million adults worldwide were obese [4]. In 2019, the International Diabetes Federation estimated that 463 million people worldwide have diabetes and predicted > 700 million cases by 2045 [3, 5]. These two diseases often occur together, and the term “diabesity” describes the pathophysiological link between them. The presence of diabesity increases the risk of developing cardiovascular disease, morbidity, and mortality [3].

Understanding the MGBA is important to clarify the origins of metabolic diseases.

## Microbiota-gut-brain axis: communication pathways

**Fig. 1:**
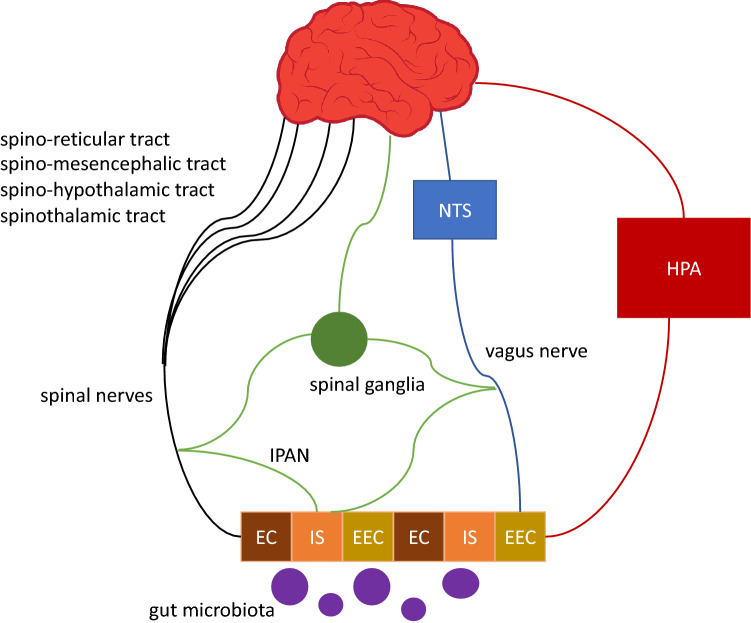
The microbiota-gut-brain axis uses several pathways that include interactions between the microbiota and the autonomic nervous system (ANS), enteric nervous system (ENS), and spinal nerves. Enteroendocrine cells (EEC) and cells of the immune system (IS), mixed with epithelial cells (EC) of the intestinal mucosa, mediate this interaction. The hypothalamic–pituitary–adrenal (HPA) axis is one of the major non-neuronal pathways used by the gut microbiota to communicate with the brain

The MGBA acts through specific communication pathways in controlling gut functions (e.g., gut motility and secretion), activating local immune responses, modulating CNS functions (e.g., mood, and psychiatric conditions associated with stress and memory), and controlling metabolism [6]. The most studied MGBA pathways include the autonomic nervous system (ANS), enteric nervous system (ENS), spinal nerve pathways, hypothalamic–pituitary–adrenal (HPA) axis, immune system, enteroendocrine cells (EEC), and microbial metabolites (Fig. 1).

1. *Autonomic nervous system*

The ANS is a network of neurons from the CNS and the peripheral nervous system (PNS). It is divided into a sympathetic and a parasympathetic branch. The vagus nerve (VN) is the major element of the parasympathetic component of the ANS. The function of ANS is to establish and regulate host physiological homeostasis, through modulation of endocrine, motor, autonomic, and behavioral responses [1]. Through the ANS, each component of the MGBA communicates bidirectionally, through complex positive and negative feedback loops.

2. *Enteric nervous system*

The ENS coordinates intestinal functions such as motility and fluid movement control. The ENS afferent fibers innervating the intestinal mucosa are derived mainly from primary intrinsic afferent neurons which form synapses with intestinal epithelial cells and EECs [7]. The information obtained from the primary intrinsic afferent neurons follows spinal and vagal afferent pathways, and arrives at the sympathetic ganglia and subsequently the CNS. As consequence, efferent pathways involving the ANS are activated. In this way, the ENS is directly associated with emotional arousal and central autonomic brain circuits, and vice versa [1]. Physiological and psychological stressors increase sympathetic tone and decrease parasympathetic tone, thereby regulating ENS operation. ENS neurons in turn innervate visceral smooth muscle and mucosal epithelial cells, thus influencing intestinal motility and secretion, mucosal permeability, immune cell functions, inflammatory reactions, and the microbiota [7].

3. *Spinal nerve pathways*

Two neuroanatomical pathways in MGBA signaling involve the pelvic nerve, which mediates non-pain functions (e. g. satiety, distention, and motility), like the VN, and the splanchnic innervation of the spinal cord, which carries sensory pain stimuli [2].

The afferent nerves of the spinal cord receive information from the viscera through sensory endings in organ walls. These nerves express a wide range of chemical and mechanosensitive receptors that are the main targets of intestinal peptides released by EECs. Like the VN, the spinal afferent nerves have collateral branches innervating the ENS. Consequently, they can perceive microbiota signals indirectly through the interaction between EEC and the ENS and transfer the corresponding intestinal information to the CNS [7].

4. *Hypothalamic–pituitary–adrenal axis*

The HPA axis is the neuroendocrine coordinator of the stress response. Its primary function is to prepare the body for the “fight or flight” response [1]. A potential link between the gut microbiota and the neuroendocrine system is suggested by disorders such as depression and irritable bowel syndrome (IBS). Increased activation of the HPA axis has been observed in depression and in IBS [5]. Furthermore, in response to stress, male germ-free mice display a hyper reactive HPA axis, thus suggesting an important role of the microbiota in HPA regulation [8]. Patients with IBS have exaggerated ACTH and cortisol responses to corticotropin releasing hormone infusion, and show altered microbiota [9, 10]. Thus, the microbiome is implicated in the regulation of the HPA axis, and vice versa. Long-term elevations in cortisol levels negatively affect gut microbiota function, by changing its composition and increasing gastrointestinal permeability. The resulting dysbiosis and bacterial translocation contribute to the chronic low-grade systemic inflammation of IBS and depression, thus inducing activation of the HPA axis [5]. Interestingly, the HPA axis interacts with the VN. In rodents, vagal stimulation increases corticotropin releasing hormone mRNA expression in the hypothalamus [11].

5. *Immune system*

The immune system has a fundamental role in regulating the symbiotic relationship between the microbiota and the host. It allows for proper interaction between microbiota and intestinal mucosa through the recognition of microbial proteins as self-antigens. In contrast, the gut microbiota responds by producing proinflammatory cytokines (e.g., IL-1, IL-6, or TNF alpha) that protect the host from pathogens [1, 12]. An innate immune signaling complex called the “inflammasome” is activated and assembled to protect against potentially pathogenic agents. Once activated, active pro-inflammatory cytokines (e.g., IL-18 and IL-1b) are produced, which in turn induce cell death through various mechanisms, thus maintaining intestinal homeostasis. Inflammasome activation has been associated with neuroinflammatory conditions and appears to play an essential role in the progression of several neurological disorders, such as multiple sclerosis, Alzheimer’s disease, and Parkinson’s disease [2].

6. *Enteroendocrine cells*

EECs account for only 1% of the epithelial cells in the gastrointestinal tract, but they make the gut the largest endocrine organ in the body [13]. They originate from the same pluripotent stem cells as well as the other cell lines of the intestinal epithelium. Moreover, EECs are arranged between the other cell lines throughout the gastrointestinal epithelium [7].

ECCs in the stomach secrete the orexigenic hormone ghrelin, gastrin, histamine, and somatostatin which control their own secretion via feedback. The EECs in the distal intestine secrete glucagon-like peptide-1 (GLP-1), peptide YY (PYY), neurotensin (NTS), oxyntomodulin (OXM), and cholecystokinin (CCK), which are anorectics. They also produce glucose-dependent insulinotropic peptide (GIP), which has an incretin effect like that of GLP-1, the motility hormones (motilin and serotonin), and other hormones such as secretin (SCT) and GLP-2, which regulate digestion and intestinal homeostasis [13]. Enteroendocrine L cells (ELs) and enterochromaffin cells (ECs) are the best-studied EECs [1]. ELs secrete GLP-1 and PYY in the postprandial phase. ECs produce most of the 5-HT in the body by using dietary tryptophan [7].

The hormones secreted by EECs engage their receptors in locally distributed cells, such as EECs, myofibroblasts and adjacent immune cells, but also interact with target organs and enteric and vagal afferent nerves. Thus, intestinal hormones appear to have different mechanisms. First, they act through paracrine signaling, given the short half-lives of many gut hormones. However, GLP-1 and GIP also operate via a distant endocrine signaling pathway. In fact, their receptors are expressed on pancreatic beta cells and in some areas of the cortex regulating food intake [13]. Furthermore, a third signaling pathway has been hypothesized, which uses the direct synaptic connection between the EEC and intestinal nerve afferent nerves. The intestinal afferent nerves of the CNS, ANS, and ENS cannot directly detect luminal chemicals. They form synaptic connections with EECs, thereby allowing sensory stimuli from the intestinal lumen to be transduced by neurotransmitters such as glutamate [14].

7. *Microbial metabolites*

The microbiota synthesizes several key neurotransmitters involved in regulating host mood, behavior, and cognitive function. It also produces branched chain amino acids, which participate in a variety of biochemical functions in the CNS and PNS [1]. The influence of the gut microbiota on specific brain circuits can help to understand unclear aspects, such as the interindividual variability of motivation to perform physical activity in mouse models [15]. Microbiome-dependent endocannabinoid production stimulates afferent sensory neuron activity and elevates dopamine levels in the ventral striatum during exercise improving performance. Conversely, alteration of the gut microbiota nullifies exercise capacity in the same manner as peripheral endocannabinoid receptor inhibition, ablation of spinal afferent neurons, or dopamine blockade [15].

Furthermore, bile acids (BAs) are intestinal microbial metabolites playing a major role in the MGBA. BAs facilitate the absorption of dietary lipids and fat-soluble vitamins from the intestinal lumen. BAs activate the nuclear farnesoid X receptor (FXR) and Takeda G protein-coupled receptor 5 (TGR5), and regulate the systemic metabolism of lipids, cholesterol, and glucose, as well as energy and immune homeostasis [1].

The most examined intestinal microbial metabolites are short-chain fatty acids (SCFAs), more than 95% of which are acetate, propionate, and butyrate. The primary source of SCFAs is non-digestible host dietary fiber that is fermented by the gut microbiota. Once absorbed into the circulation, SCFAs serve as energy substrates, influence the maturation of microglia in the CNS, and act as CNS signaling molecules [12, 14].

SCFAs bind G protein-coupled receptors, the most studied of which are free fatty acid receptors 2 (FFAR2, also known as GPR43) and 3 (FFAR3, also known as GPR41). FFAR2 is expressed in adipocytes and skeletal muscle, and FFAR3 is expressed in the PNS, blood brain barrier, the colon, immune cells, and the heart [1]. Butyrate also activates the olfactory receptor of family 51 subfamily E member 1 (OR51E1). Acetate and propionate activate the olfactory receptor family subfamily 2 member 51 (OR51E2), which have been detected in the brains of rodents but not in humans [16].

SCFAs have many effects, among which the best known are epigenetic. Notably, all have histone deacetylase inhibitory effects, but butyrate is the most potent inhibitor of class I and IIa histone deacetylase. Furthermore, acetate can be converted to acetyl-CoA, thus increasing histone acetylation [1]. FFAR2, FFAR3, and OR51E1 are expressed by colonic ELs. Their activation causes the secretion of GLP-1 and PYY. SCFAs also stimulate the secretion of insulin, ghrelin, leptin, and amylin [12]. Thus, they slow gastric emptying and intestinal transit by increasing energy absorption and glucose-dependent insulin release. Furthermore, SCFAs indirectly affect appetite and food intake via systemic circulation and vagal afferent nerves, stimulate ECs to produce serotonin, and additionally affect intestinal motility [17].

## Roles of MGBA in regulating appetite and glucose homeostasis

**Fig. 2:**
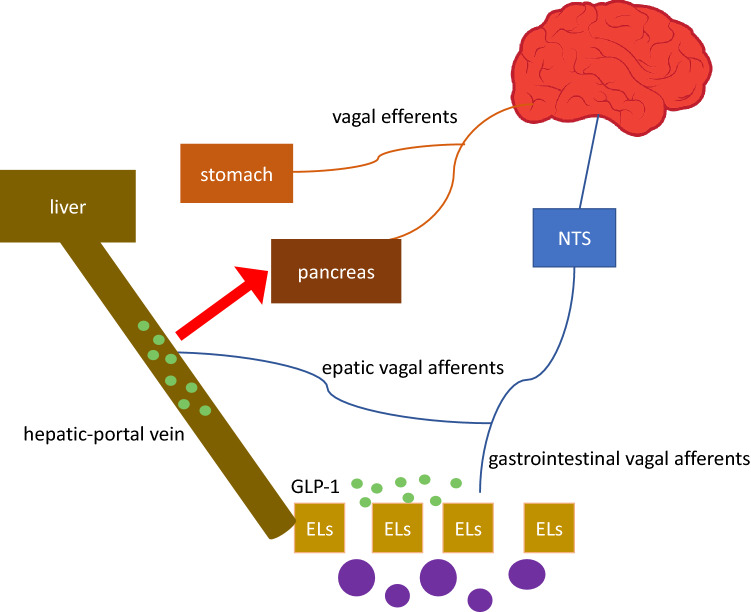
GLP-1 secretion stimulated by meal and gut microbiota. GLP-1 binds specific receptors on gastrointestinal and hepatic vagal afferents transmitting information to the nucleus of the solitary tract (NST) of the brainstem and higher brain centers. From here, the vagal efferents depart to innervate stomach and pancreas. This circuit allows to control gastric emptying and food intake, reinforcing the direct incretin effect of GLP-1 on the pancreas

The role of MGBA as a central regulator of metabolism and appetite is increasingly evident. The main players in the appetite control pathway are the intestinal hormones produced by the EECs, the VN, the hypothalamus, and the brainstem. The brain receives hormonal and vagal signals, which carry information from the periphery and the intestinal lumen components, including the microbiota [18]. Because of its EEC-mediated interaction with the gut microbiota, the VN is a potential pathway through which gut microorganisms influence host feeding behavior [19] (Fig. 2).

1. *Intestinal hormones*

Before ingestion of food, gastric EECs produce the hormone orexigenic ghrelin, which uses a vagal circuit involving the hindbrain and the hypothalamic arcuate nucleus (ARC) to increase appetite [13]. Afterward, nutrients absorbed across the duodenal epithelium bind ECC receptors and stimulate them to secrete several hormones, including CCK, GIP, and SCT. CCK receptors are expressed by vagal afferent nerves. Once activated, they transfer information to the nucleus of the solitary tract, thereby contributing to satiety. Furthermore, CCK acts on the vagal endings by inducing contraction of the pyloric sphincter and slowing gastric emptying. GIP promotes insulin secretion by activating specific receptors on beta cells and induces satiety by acting on the hypothalamus. SCT promotes the secretion of pancreatic enzymes, but its role in brain signaling is not fully clarified [13].

After transitioning from the duodenum to the jejunum, nutrients and bile acids activate intestinal EECs to secrete GLP-1, PYY, NTS, CCK, GIP, and SCT. The receptors of these hormones, except for that of GIP, are found on the vagal afferent nerves. Furthermore, GLP-1, PYY, and CCK receptors are localized in the ENS terminals. GLP-1, GIP, PYY, and NTS receptors are also located in the central feeding centers. The activation of all receptors contributes to satiety [13].

2. *GLP-1*

GLP-1 is a 30 amino acid peptide derived from preproglucagon. Two bioactive forms of GLP-1 exist, GLP-17e37 and GLP-17e36 amide, which are secreted by Els in response to an oral glucose load [20]. In the large intestine, the gut microbiota uses undigested dietary nutrients, fiber, and bile acids to produce metabolites such as SCFAs, indoles, and BAs, which induce further secretion of GLP-1 from ELs [13]. In the proximal gut, luminal nutrients induce the postprandial peak of GLP-1 and PYY; in the distal gut, their release is activated almost exclusively by the gut microbiota via its membrane components such as lipopolysaccharide or metabolites [7], thus sustaining the secretion of GLP-1 and PYY for many hours after a meal [1].

GLP-1 acts on insulin secretion and satiety: it increases insulin and decreases glucagon secretion, thereby blocking the endogenous production of glucose and decreasing glycemia after a meal. GLP-1 also delays gastric emptying [20] and increases pyloric contraction, thus decreasing the flow of food into the small intestine [13]. Furthermore, GLP-1 receptors (GLP-1Rs) are centrally expressed in the hindbrain, hypothalamus, hippocampus, and mesolimbic system, and contribute to appetite regulation. Indeed, administering GLP-1R agonists into the fourth ventricle in mice decreases food intake and body weight [21, 22], and GLP-1R knockdown in NTS neurons increases food intake [23, 23]. In adipose and muscle tissue, GLP-1 and GLP-1R agonists (i.e., exenatide) have an insulin-like effect by stimulating intracellular glucose transport via phosphorylation of AMP-activated protein Kinase (AMPK) and the subsequent translocation of glucose transporter 4 (Glut-4) into the plasma membrane [24]. GLP-1R agonists also exhibit cytoprotective and proliferative effects on beta, alpha and delta cells. Chronic administration of exenatide increases cell replication, reduces apoptosis and cellular stress, and increases insulin sensitivity in a nonhuman primate [25]. Exenatide may promote differentiation of pancreatic ductal cells to a beta-cell phenotype in baboons without inducing pancreatitis, accumulation of parenchymal or periductal inflammatory cells, ductal hyperplasia, dysplastic lesions, or pancreatic intraepithelial neoplasia [26].

However, endogenous GLP-1 is rapidly degraded by dipeptidyl peptidase-4 (DPP-4). Thus, an additional mechanism exists through which the GLP1 signal is transmitted from the periphery to the center. The VN and the brainstem VN have been hypothesized to mediate this communication [20]. Peripheral administration of GLP-1 activates brainstem neurons in rats [27], but the anorectic effects of GLP-1 disappear after vagotomy in rodents [27, 28]. A study in patients with bilateral total subdiaphragmatic vagotomy and pyloroplasty has demonstrated that, even in humans, an intact VN is required for intravenously administered GLP-1 to have anorectic effects [29]. Studies on subdiaphragmatic vagal deafferentation, which spares approximately 50% of vagal afferent nerves, have also confirmed the roles of vagal afferent nerves in mediating the effects of GLP-1 [30, 31], as confirmed in a mouse model with GLP-1R knockdown in vagal afferent nerves [32]. In conclusion, GLP-1 exerts its anorectic effect by activating vagal GLP-1Rs.

VN and GLP-1 are also linked through another type of interaction called the neuroincretin effect. The incretin effect of GLP-1 is largely due to its direct actions on pancreatic beta and delta cells, but another vagal signaling pathway may exist that enhances incretin effects [33]. Activation of vagal afferent nerves has been observed after GLP-1 administration [34], whereas GLP-1 administration has been found not to suppress postprandial insulin secretion in patients with bilateral total subdiaphragmatic vagotomy and pyloroplasty [29]. Furthermore, glucose uptake from the gastrointestinal tract is impaired in patients with bilateral total subdiaphragmatic vagotomy and pyloroplasty [35], thus suggesting a role of vagal mediation in GLP-1’s incretin effect.

This aspect can be extended to the entire MGBA. In a recent study, mice fed a high-fat diet were exposed to butyrate, intragastrically or via intravenous injection. Intragastric butyrate decreased food intake, whereas intravenous injection did not lead to any changes. These findings suggest that the effect of butyrate on feeding behavior is mediated by a mechanism involving gut-brain neural circuits [36]. Supporting this hypothesis, a study has demonstrated that modifying the composition of the gut microbiome with prebiotics affects portal GLP-1 levels, which in turn affects food intake [37]. Furthermore, in GLP-1R knockout mice, prebiotic treatment does not elicit the same beneficial effects [38–40].

3. *Vagus nerve*

The VN is the tenth cranial nerve. It has an afferent sensory branch (80% fibers) and an efferent motor component (20% fibers). The intestinal vagal innervation is divided into two branches: the hepatic branch, which innervates the hepatoportal bed, and the celiac branch [1]. The VN tonically transmits crucial information from gastrointestinal, respiratory, and cardiovascular systems, and provides feedback responses [1].

Given their expression of a large variety of receptors, vagal afferent nerves can respond to a wide variety of mechanical, chemical, or hormonal signals [1]. Some intestinal vagal afferent nerves are intraganglionic laminar endings and act as mechanoreceptors detecting intestinal distension [13]. The rest of th vagal afferent nerves reach the mucosal layer and the myenteric layer near the EEC, mucosal immune cells, and ENS neurons. These nerves act like chemoreceptors and do not come into direct contact with the intestinal endoluminal substances. Thus, they form synapses with the intestinal epithelial cells and the EECs [1]. In fact, evidence suggests that synaptic structures connect vagal chemoreceptors and a basolateral cytoplasmic process of the ECCs called the neuropod [13]. Synapses rapidly transduce signals from the intestinal lumen to vagal neurons across glutamatergic and serotonergic signals [41]. Vagal chemoreceptors appear to modulate the relationship between the microbiota and the brain. The activation of vagal afferent nerves by microorganisms has been demonstrated by injection of colonies of Lactobacillus johnsonii into the duodenum, thus resulting in improvement in gastric vagal activity [42]. Furthermore, activation of the NTS has been observed after oral administration of Campylobacter jejuni in mice [43].

As described before, beyond the synaptic structure, vagal afferent nerves receive information through paracrine and endocrine signaling pathways [13]. In fact, GLP-1Rs are expressed on both the hepatic and the intestinal vagal branches [33]. Thus, the effects of intestinal GLP-1 may be mediated by GLP-1R activation on hepatoportal vagal fibers (through the endocrine pathway) or on intestinal vagal afferent nerves (through paracrine stimulation).

The cell bodies of vagal afferent nerves are found in the nodose ganglia. From there, vagal fibers project to the nucleus of the solitary tract, and to the area postrema and dorsal motor nucleus [1].

4. *Hypothalamus and brainstem*

The hypothalamic ARC plays a crucial role in regulating appetite. It consists of two populations of neurons with opposing effects. Orexigenic neurons secrete neuropeptide Y and agouti-associated protein. Anorexigenic neurons produce pro-opiomelanocortin-derived alpha-melanocyte stimulating hormone and cocaine-amphetamine regulated peptide. The ARC is supplied by fenestrated capillaries, through which the intestinal hormones directly act on the neurons of the ARC. In turn, the ARC projects to several extra-hypothalamic and intra-hypothalamic regions, including the paraventricular hypothalamic nucleus, where efferent pathways that regulate energy expenditure begin [20]. Furthermore, the NTS receives information from intestinal mechanoreceptive and chemoreceptive vagal afferent nerves and sends fibers to the hypothalamus [20].

The intestinal microbiota influences hypothalamic activity and the brainstem. The increased diversity of the gut microbiota significantly correlates with the sparing of the hypothalamic microstructure in obese and non-obese individuals [44] Furthermore, in animal models, the gut microbiota modulates hypothalamic gene expression, neuropeptide secretion, neurotransmitter levels, and neuronal activity [45, 46]. SCFA administration decreases energy intake in mice and humans, both directly, by affecting central neurons, and indirectly, through peripheral circuits innervating the hypothalamus [47–49].

The VN provides a potential link between the gut microbiota and the brainstem, by modulating NTS function and downstream projection sites. A high fat diet alters the microbial composition and causes microglial activation and vagal afferent reorganization in the NTS [50, 51].

## Roles of the MGBA in the development of obesity and diabetes

Obesity and T2DM are metabolic disorders whose prevalence has significantly increased in recent decades. These pathologies have important public health implications affecting both industrialized countries and developing continents such as Africa and Asia. It also occurs in the European countries that rapidly transitioned from a totalitarian regime to a free and oriented economy [52]. In this scenario, the classic risk factors are joined by others linked to the evolution of economic pressure and consumer demand. For instance, shift work increases the risk of several chronic disturbances, such as T2D and cardiovascular disease [53].

The term “diabesity” defines the pathophysiological connection between overweight and T2DM [54]. MGBA, through the gut microbiota, VN and GLP-1, has been hypothesized to have a key pathophysiological role in the development of metabolic disorders and neurological pathologies and represents a connection between them [55].

Insulin resistance (IR), the basis of the pathophysiology of diabesity, has a genetic basis which is involved in CVD risk [56]. Several variants of genes involved in the modulation of insulin action have been described. Among them, single nucleotide polymorphisms (SNPs) of rs1044498 (i.e., ENPP1 K121Q), rs1801278 (i.e., IRS1 G972R) and rs2295490 (TRIB3 Q84R) genes showed a combined effect on major CVDs in high-risk individuals, through their ability to influence the IR both whole body and at the endothelial level [56]. Endothelial IR is implicated in the progression of atherosclerosis. Thus, defective insulin signaling appears to be the molecular basis of the pathogenic role played by IR on CVD [56].

The effect of IR occurs at two levels: peripheral and central. At the peripheral level, a lack of response of liver, skeletal muscle, and adipose tissue to the hormones released after increased glycemia is observed. At the central level, disturbances in cerebral mitochondrial function and cellular insulin signaling are observed. In particular, the phosphoinositide-3 kinase (PI3K-PKB)/Akt pathway and the Ras/mitogen-activated kinase (MAPK) are involved [54, 57], thus affecting cell survival, energy metabolism, synaptic plasticity, and memory and learning processes.

Furthermore, insulin controls hepatic glucose production and glucose homeostasis through binding specific hepatic and brain receptors. In the liver, insulin activates STAT3 transcription factors in hepatocytes that suppress the gene expression of gluconeogenic enzymes, thereby decreasing hepatic glucose production. In the hypothalamus, STAT3 binds specific receptors on the ARC, thus inducing hyperpolarization of hypothalamic neurons by stimulation of the VN [58]. The VN is indispensable in the central action of insulin on the control of hepatic glucose production. If the hepatic branch of the VN is excised, the insulin-induced suppression of hepatic glucose production is attenuated [59]. Moreover, nerve activity is enhanced by an increase in blood sugar and is decreased by an increase in plasma insulin concentration [60]. Furthermore, mice develop insulin resistance after vagotomy [61].

The MGBA may be implicated in IR through not only the VN but also the close association of IR with the gut microbiota. In humans, as also observed in mouse models, transplantation of the gut microbiome of healthy people into patients with metabolic syndrome increases the insulin sensitivity of the recipients [62]. Furthermore, endotoxemia produced by intestinal dysbiosis may trigger the low-grade inflammation observed in obesity and IR [63].

Dysregulation of membrane proteolysis of IR-linked pro-inflammatory Tumor Necrosis Factor Alpha (TNF-α) by TNF-α converting enzyme (TACE)/metalloproteinase 3 (TIMP3) system has been proposed as a common feature of glucose intolerance and vascular inflammation [64]. Heterozygosity for the insulin receptor null allele (Insr + /–) causes down-regulation of Timp3 that results in uncontrolled protease activity of TACE, thereby increasing circulating soluble TNF-α levels. The resulting uncontrolled inflammatory state interacts with the haploinsufficiency of Insr, leading to glucose intolerance and vascular inflammation [65]. Moreover, defects in glucose and fatty acid metabolism result in intracellular accumulation of fatty acids and glycolytic intermediates. As a result, interferences with insulin signaling begin such as impairment of mitochondrial function and muscle oxidative capacity, and activation of molecular inflammatory pathways (i.e., protein kinase C, nuclear factor kB, and toll-like receptor 4 (TLR4) networks) [66]. Changes in the gut microbiota may reduce the integrity of the intestinal barrier leading to increased leakage of lipopolysaccharides and fatty acids that act on TLR4 to activate systemic inflammation. Fatty acids can also trigger endoplasmic reticulum stress, which can be further stimulated by cross talk with active TLR4 [67]. Studies have shown that antidiabetic drugs such as pioglitazone significantly reduce the enzyme activity of skeletal muscle TNF-α and TACE and improve the mitochondrial proteomic profile of skeletal muscle in subjects with T2D [68].

Obese people have lower microbial diversity than people of normal weight, thus resulting in a different pool of microbial metabolites with different influences on energy homeostasis and GLP-1 secretion [69]. In obese mice, microbiota imbalance decreases the expression of GLP-1Rs. Although the use of GLP-1R agonists effectively decreases HbA1c, not all patients respond to treatment, and a drug-resistant state may occur. The mechanisms responsible for the unresponsiveness to GLP-1 may be associated with alterations in the MGBA. A high-fat diet has been shown to alter the gut microbiota composition and to induce GLP-1 resistance by impairing nitric oxide production in enteric neurons and consequently attenuating gut-brain signaling [70]. Notably, liraglutide treatment and bariatric surgery change the gut microbial composition to a profile similar to that observed in lean mice [70, 72]. In humans, prebiotic treatment increases microbiome diversity, and consequently GLP-1 levels and satiety [73, 74].

In addition, VN responsiveness appears to be impaired in obesity. Vagal neurocircuits have a plasticity that allows them to generate various phenotypes in different conditions [75, 76]. During fasting, when circulating CCK levels are low, the density of cannabinoid and melanin concentration hormone receptors on vagal afferent nerves increases. This “orexigenic phenotype” is associated with an increased sense of hunger. After food intake, circulating CCK levels and neuropeptide Y receptor expression in vagal afferent nerves increase, whereas the level of cannabinoid receptors decreases. This “anorexigenic phenotype” is associated with a decrease in hunger. In obesity, vagal afferent nerves appear to show an orexigenic phenotype regardless of feeding status [75].

## Conclusion

The roles of gut hormones, the VN and the MGBA in appetite regulation and metabolic control are receiving increasing interest. Obesity and T2DM have had strong economic and socio-health effects in recent years [77]. Common pathophysiological mechanisms link these pathologies and may be based on impaired insulin signaling and MGBA integrity. Thus, the MGBA may serve as a potential target for specific antidiabetic therapies. Specifically, the VN, the pathway through which gut microorganisms influence host feeding behavior, warrants further investigation.

## References

[1] Cryan JF, O’Riordan KJ, Cowan CSM (2019). The microbiota-gut-brain axis. Physiol Rev.

[2] Rutsch A, Kantsjö J B, Ronchi F, (2020). The gut-brain axis: how microbiota and host inflammasome influence brain physiology and pathology. Frontiers in Immunology..

[3] Tilinca MC, Tiuca RA, Niculas C (2021). Future perspectives in diabesity treatment: Semaglutide, a glucagon-like peptide 1 receptor agonist (Review). Exp Ther Med..

[4] World Health Organization (WHO): Obesity and Overweight, 2020 https://www.who.int/news‐room/fact‐sheets/detail/obesity‐ and‐overweight. Accessed April 21, 2021.

[5] Farzi A, Fröhlich E, Holzer P (2018). Gut microbiota and the neuroendocrine system. Neurotherapeutics.

[6] Browning KN, Verheijden S, Boeckxstaens GE (2017). The vagus nerve in appetite regulation, mood and intestinal inflammation. Gastroenterology.

[7] Kuwahar A, Matsud K, Kuwahara Y (2020). Microbiota-gut-brain axis: enteroendocrine cells and the enteric nervous system form an interface between the microbiota and the central nervous system. Biomed Res.

[8] Sudo N, Chida Y, Aiba Y (2004). Postnatal microbial colonization programs the hypothalamic-pituitary-adrenal system for stress response in mice. J Physiol.

[9] Eisenstein M (2016). Microbiome: bacterial broadband. Nature.

[10] Grenham S, Clarke G, Cryan JF (2011). Brain-gut-microbe communication in health and disease. Front Physiol.

[11] Hosoi T, Okuma Y, Nomura Y (2000). Electrical stimulation of afferent vagus nerve induces IL-1beta expression in the brain and activates HPA axis. Am J Physiol Regul Integr Comp Physiol.

[12] Doroszkiewicz J, Groblewska M, Mroczko B (2021). The role of gut microbiota and gut–brain interplay in selected diseases of the central nervous system. Int J Mol Sci.

[13] Richards P, Thornberry NA, Pinto S (2021). The gut-brain axis: Identifying new therapeutic approaches for type 2 diabetes, obesity, and related disorders. Mol Metab..

[14] Heiss CN, Olofsson LE (2019). The role of the gut microbiota in development, function and disorders of the central nervous system and the enteric nervous system. J Neuroendocrinol..

[15] Dohnalová L, Lundgren P, Carty JRE (2022). A microbiome-dependent gut-brain pathway regulates motivation for exercise. Nature.

[16] Yuan TT, Toy P, McClary JA, Lin RJ, Miyamoto NG, Kretschmer PJ (2001). Cloning and genetic characterization of an evolutionarily conserved human olfactory receptor that is differentially expressed across species. Gene.

[17] Portincasa P, Bonfrate L, Vacca M (2022). Gut microbiota and short chain fatty acids: implications in glucose homeostasis. Int J Mol Sci.

[18] Aydin Ö, Nieuwdorp M, Gerdes V (2018). The gut microbiome as a target for the treatment of type 2 diabetes. Curr DiabRep.

[19] Bonaz B, Bazin T, Pellissier S (2018). The vagus nerve at the interface of the microbiota-gut-brain axis. Front Neurosci.

[20] Sam AH, Troke RC, Tan TM, Bewick GA (2012). The role of the gut/brain axis in modulating food intake. Neuropharmacology.

[21] Hayes MR, Skibicka KP, Grill HJ (2008). Caudal brainstem processing is sufficient for behavioral, sympathetic, and parasympathetic responses driven by peripheral and hindbrain glucagon like-peptide-1 receptor stimulation. Endocrinology.

[22] Richard JE, Anderberg RH, Göteson A, Gribble FM, Reimann F, Skibicka KP (2015). Activation of the GLP-1 receptors in the nucleus of the solitary tract reduces food reward behavior and targets the mesolimbic system. PLoS One.

[23] Alhadeff AL, Mergler BD, Zimmer DJ (2017). Endogenous glucagon-like peptide-1 receptor signaling in the nucleus tractus solitarius is required for food intake control. Neuropsychopharmacology..

[24] Alhadeff AL, Grill HJ (2014). Hindbrain nucleus tractus solitarius glucagon-like peptide-1 receptor signaling reduces appetitive and motivational aspects of feeding. Am J Physiol Integr Comp Physiol.

[25] Andreozzi F, Raciti GA, Nigro C (2016). The GLP-1 receptor agonists exenatide and liraglutide activate Glucose transport by an AMPK-dependent mechanism. J Transl Med..

[26] Fiorentino TV, Casiraghi F, Davalli AM (2019). Exenatide regulates pancreatic islet integrity and insulin sensitivity in the nonhuman primate baboon Papio hamadryas. JCI Insight.

[27] Fiorentino TV, Owston M, Abrahamian G (2015). Chronic continuous exenatide infusion does not cause pancreatic inflammation and ductal hyperplasia in non-human primates. Am J Pathol.

[28] Imeryuz N, Yegen BC, Bozkurt A, Coskun T, Villanueva-Penacarrillo ML, Ulusoy NB (1997). Glucagon-like peptide-1 inhibits gastric emptying via vagal afferent-mediated central mechanisms. Am J Physiol.

[29] Abbott CR, Monteiro M, Small CJ (2005). The inhibitory effects of peripheral adminis- tration of peptide YY(3–36) and glucagon-like peptide-1 on food intake are attenuated by ablation of the vagal-brainstem-hypothalamic pathway. Brain Res..

[30] Plamboeck A, Veedfald S, Deacon CF (2013). The effect of exogenous GLP-1 on food intake is lost in male truncally vagotomized subjects with pyloroplasty. AJP Gastrointest Liver Physiol.

[31] Labouesse MA, Stadlbauer U, Weber E, Arnold M, Langhans W, Pacheco-López G (2012). Vagal afferents mediate early satiation and prevent flavour avoidance learning in response to intraperitoneally infused exendin-4. J Neuroendocrinol.

[32] Ruttimann EB, Arnold M, Hillebrand JJ, Geary N, Langhans W (2008). Intrameal hepatic portal and intraperitoneal infusions of glucagon-like peptide-1 reduce spontaneous meal size in the rat via different mechanisms. Endocrinology.

[33] Krieger JP, Arnold M, Pettersen KG, Lossel P, Langhans W, Lee SJ (2016). Knockdown of GLP-1 receptors in vagal afferents affects normal food intake and glycemia. Diabetes.

[34] Krieger JP, Langhans W, Lee SJ (2015). Vagal mediation of GLP-1’s effects on food intake and glycemia. Physiol Behav.

[35] Nakabayashi H, Nishizawa M, Nakagawa A, Takeda R, Niijima A (1996). Vagal hepatopancreatic reflex effect evoked by intraportal appearance of tGLP-1. Am J Phys..

[36] Plamboeck A, Veedfald S, Deacon CF (2013). Characterisation of oral and i.v. glucose handling 760 in truncally vagotomised subjects with pyloroplasty. Eur J Endocrinol..

[37] Li Z, Yi C-X, Katiraei S (2018). Butyrate reduces appetite and activates brown adipose tissue via the gut-brain neural circuit. Gut.

[38] Cani PD, Dewever C, Delzenne NM (2007). Inulin-type fructans modulate gastrointestinal peptides involved in appetite regulation (glu- cagon-like peptide-1 and ghrelin) in rats. Br J Nutr.

[39] Cani PD, Knauf C, Iglesias MA, Drucker DJ, Delzenne NM, Burcelin R (2006). Improvement of glucose tolerance and hepatic insulin sensitivity by oligofructose requires a functional Glucagon-Like Peptide 1 Receptor. Diabetes.

[40] Samuel BS, Shaito A, Motoike T (2008). Effects of the gut microbiota on host adiposity are modulated by the short-chain fatty-acid binding G protein-coupled receptor, Gpr41. Proc Natl Acad Sci U S A.

[41] Cani PD, Lecourt E, Dewulf EM (2009). Gut microbiota fermentation of prebiotics increases satietogenic and incretin gut peptide production with consequences for appetite sensation and glucose response after a meal. Am J Clin Nutr.

[42] Yu KB, Hsiao EY (2021). Roles for the gut microbiota in regulating neuronal feeding circuits. J Clin Invest..

[43] Tanida M, Yamano T, Maeda K, Okumura N, Fukushima Y, Nagai K (2005). Effects of intraduodenal injection of Lactobacillus johnsonii La1 on renal sympathetic nerve activity and blood pressure in urethane-anesthetized rats. Neurosci Lett.

[44] Gaykema RP, Goehler LE, Lyte M (2004). Brain response to cecal infection with Campylobacter jejuni: analysis with Fos immunohistochemistry. Brain Behav Immun.

[45] Fernandez-Real JM, Serino M, Blasco G (2015). Gut microbiota interacts with brain microstructure and function. J Clin Endocrinol Metab..

[46] Schéle E, Grahnemo L, Anesten F, Hallén A, Bäckhed F, Jansson JO (2013). The gut microbiota reduces leptin sensitivity and the expression of the obesity- suppressing neuropeptides proglucagon (Gcg) and brain-derived neurotrophic factor (Bdnf) in the central nervous system. Endocrinology.

[47] Yao H, Fan C, Fan X (2020). Effects of gut microbiota on leptin expression and body weight are lessened by high- fat diet in mice. Br J Nutr.

[48] Blaak EE, Canfora EE, Theis S (2020). Short chain fatty acids in human gut and metabolic health. Benef Microbes.

[49] Frost G, Sleeth ML, Sahuri-Arisoylu M (2014). The short-chain fatty acid acetate reduces appetite via a central homeostatic mechanism. Nat Commun.

[50] De Vadder F, Kovatcheva-Datchary P, Goncalves D, Vinera J, Zitoun C, Duchampt A (2014). Microbiota-generated metabolites promote metabolic benefits via gut-brain neural circuits. Cell.

[51] Vaughn AC, Cooper EM, Di Lorenzo PM (2017). Energy-dense diet triggers changes in gut microbiota, reorganization of gut-brain vagal communication and increases body fat accumulation. Acta Neurobiol Exp (Wars).

[52] Minaya DM, Turlej A, Joshi A (2020). Consumption of a high energy density diet triggers microbiota dysbiosis, hepatic lipidosis, and microglia activation in the nucleus of the solitary tract in rats. Nutr Diabetes..

[53] Zoto E, Cenko F, Doci P, Rizza S (2019). Effect of night shift work on risk of diabetes in healthy nurses in Albania. Acta Diabetol..

[54] Rizza S, Longo S, Piciucchi G (2020). Carotid intimal medial thickness in rotating night shift is related to IL1β/IL6 axis. Nutr Metab Cardiovasc Dis..

[55] Cardoso S, Moreira PI (2019). Diabesity and brain disturbances: a metabolic perspective. Mol Aspects Med.

[56] Farzi A, Hassan AM, Zenz G, Holzer P (2019). Diabesity and mood disorders: multiple links through the microbiota-gut-brain axis. Mol Aspects Med.

[57] Bacci S, Prudente S, Copetti M (2013). Joint effect of insulin signaling genes on cardiovascular events and on whole body and endothelial insulin resistance. Atherosclerosis.

[58] Cardoso S, Seiça R, Moreira PI (2017). Diabesity and brain energy metabolism: the case of alzheimer’s disease. Adv Neurobiol.

[59] Inoue H (2016). Central insulin-mediated regulation of hepatic glucose production [Review]. Endocr J.

[60] Pocai A, Lam TK, Gutierrez-Juarez R (2005). Hypothalamic K(ATP) channels control hepatic glucose production. Nature.

[61] Niijima A (1985). Blood glucose levels modulate efferent activity in the vagal supply to the rat liver. J Physiol.

[62] Fernandes AB, Patarrão RS, Videira PA, Macedo MP (2011). Understanding postprandial glucose clearance by peripheral organs: the role of the hepatic parasympathetic system. J Neuroendocrinol.

[63] Vrieze A, Van Nood E, Holleman F (2012). Transfer of intestinal microbiota from lean donors increases insulin sensitivity in individuals with metabolic syndrome. Gastroenterology.

[64] Cani PD, Amar J, Iglesias MA (2007). Metabolic endotoxemia initiates obesity and insulin resistance. Diabetes.

[65] Monroy A, Kamath S, Chavez AO (2009). Impaired regulation of the TNF-alpha converting enzyme/tissue inhibitor of metalloproteinase 3 proteolytic system in skeletal muscle of obese type 2 diabetic patients: a new mechanism of insulin resistance in humans. Diabetologia.

[66] Federici M, Hribal ML, Menghini R (2005). Timp3 deficiency in insulin receptor-haploinsufficient mice promotes diabetes and vascular inflammation via increased TNF-alpha. J Clin Invest.

[67] Fiorentino TV, Monroy A, Kamath S (2021). Pioglitazone corrects dysregulation of skeletal muscle mitochondrial proteins involved in ATP synthesis in type 2 diabetes. Metabolism..

[68] Velloso LA, Folli F, Saad MJ (2015). TLR4 at the crossroads of nutrients, gut microbiota, and metabolic inflammation. Endocr Rev.

[69] Tripathy D, Daniele G, Fiorentino TV (2013). Pioglitazone improves glucose metabolism and modulates skeletal muscle TIMP-3-TACE dyad in type 2 diabetes mellitus: a randomised, double-blind, placebo-controlled, mechanistic study. Diabetologia.

[70] Salehi M, Purnell JQ (2019). The role of glucagon-like peptide-1 in energy homeostasis. Metab Syndr Relat Disord.

[71] Grasset E, Puel A, Charpentier J (2017). A specific gut microbiota dysbiosis of type 2 diabetic mice induces GLP- 1 resistance through an enteric NO-dependent and gut- brain axis mechanism. Cell Metab.

[72] Magouliotis DE, Tasiopoulou VS, Sioka E, Chatedaki C, Zacharoulis D (2017). Impact of bariatric surgery on metabolic and gut microbiota profile: a systematic review and meta-analysis. Obes Surg.

[73] Wang L, Li P, Tang Z, Yan X, Feng B (2016). Structural modulation of the gut microbiota and the relationship with body weight: compared evaluation of liraglutide and saxagliptin treatment. Sci Rep.

[74] Everard A, Cani PD (2013). Diabetes, obesity and gut microbiota. Best Pract Res Clin Gastroenterol.

[75] Cani PD, Joly E, Horsmans Y, Delzenne NM (2006). Oligofructose promotes satiety in healthy human: a pilot study. Eur J Clin Nutr.

[76] Dockray GJ (2014). Gastrointestinal hormones and the dialogue between gut and brain. J Physiol.

[77] Kang YM, Bielefeldt K, Gebhart GF (2004). Sensitization of mechanosensitive gastric vagal afferent fibers in the rat by thermal and chemical stimuli and gastric ulcers. J Neurophysiol.

[78] Tilinca MC, Tiuca RA, Burlacu A, Varga AA (2021). Update on the use of liraglutide in the modern treatment of ‘diabesity’: a narrative review. Medicina (Kaunas)..

